# The Holistic Effects of Climate Change on the Culture, Well-Being, and Health of the Saami, the Only Indigenous People in the European Union

**DOI:** 10.1007/s40572-018-0211-2

**Published:** 2018-10-22

**Authors:** Jouni J. K. Jaakkola, Suvi Juntunen, Klemetti Näkkäläjärvi

**Affiliations:** 1 Center for Environmental and Respiratory Health Research, University of Oulu, P. O. Box 5000, FI-90014 Oulu, Finland; 2 Department of Social Sciences, University of Lapland, Rovaniemi, Finland

**Keywords:** Climate change, Saami people, Reindeer herding, Systematic review, Public health, Adaptation

## Abstract

**Purpose of Review:**

(1) To develop a framework for understanding the holistic effects of climate change on the Saami people; (2) to summarize the scientific evidence about the primary, secondary, and tertiary effects of climate change on Saami culture and Sápmi region; and (3) to identify gaps in the knowledge of the effects of climate change on health and well-being of the Saami.

**Recent Findings:**

The Saami health is on average similar, or slightly better compared to the health of other populations in the same area. Warming climate has already influenced Saami reindeer culture. Mental health and suicide risk partly linked to changing physical and social environments are major concerns.

**Summary:**

The lifestyle, diet, and morbidity of the Saami are changing to resemble the majority populations posing threats for the health of the Saami and making them more vulnerable to the adverse effects of climate change. Climate change is a threat for the cultural way of life of Saami. Possibilities for Saami to adapt to climate change are limited.

## Introduction

From the global perspective, the indigenous people in the Artic constitute potentially the most vulnerable population to the effects of climate change for two reasons. They live in close interaction with the natural environment and the climate change and its effects to environmental conditions including temperature are most impactful in the Arctic [1]. Therefor, indigenous people could be regarded as the first population indicators of the effects of harmful environmental condition and change. From the perspective of the indigenous people, climate change has been regarded as one of the most extensive threats to health and well-being [2]. In addition to climate change, environmental change related to natural and human-based reasons, continued regional economic development, and prolific utilization of natural resources constitute similar threats to health, well-being as well as to the entire culture.^1^

The average temperature in the Arctic has already risen from the preindustrial period [3]. The effects of climate change in the Polar Regions are expected to be globally the most pronounced [1]. Conditions in the Sápmi region have changed during postindustrial period, including the timing of snowmelt and the length of thermal seasons [3, 4].

Around 40 indigenous peoples inhabit the Arctic Region forming 10% of the total population in this region. The present article focusses on the Saami (in North Saami: *Sámi*), the indigenous people living in the northern parts of Norway, Sweden, Finland, and Kola Peninsula in Russia. Many of the phenomena related to the Saami are likely to be generalizable to the other indigenous populations. On the basis of a priori knowledge, we hypothesized that the effects of climate change are likely to be holistic, influencing not just health and well-being, but the entire culture. Our overall objective was to elaborate the holistic effects of climate change on the Saami. The specific objectives of the study were (1) to develop a framework for understanding the holistic effects of climate change on the Saami people and Arctic indigenous people in general; (2) to summarize the scientific evidence about the primary, secondary, and tertiary effects of climate change to Saami culture and to Sápmi region; and (3) to identify gaps in the knowledge of the effects of climate change on health and well-being of the Saami.

## Methods

### Saami People

The traditional settlement area is called *Sápmi* (land of the Saami) in the North Saami language. The population of Saami people is estimated at 50,000–70,000 in Norway [5], over 10,000 in Finland [6], 20,000–35,000 in Sweden [7••], and 2000 in Russia [8]. Approximately half of the Saami speak Saami as their native language. All Saami languages are classified as endangered or highly endangered [9]. Traditional Saami livelihoods include reindeer herding, fishing, hunting, handicrafts (duodji), and gathering. Reindeer herding is the most viable livelihood. Many of the Saami reindeer-herding families are also involved in Saami handicrafts, fishing, and gathering. In Sweden and Norway, practically only the Saami can practice reindeer herding. At present many Saami are occupied outside traditional livelihoods and many, especially youth, have migrated to urban regions outside Sápmi [10].

### Framework and Theory

**Fig. 1:**
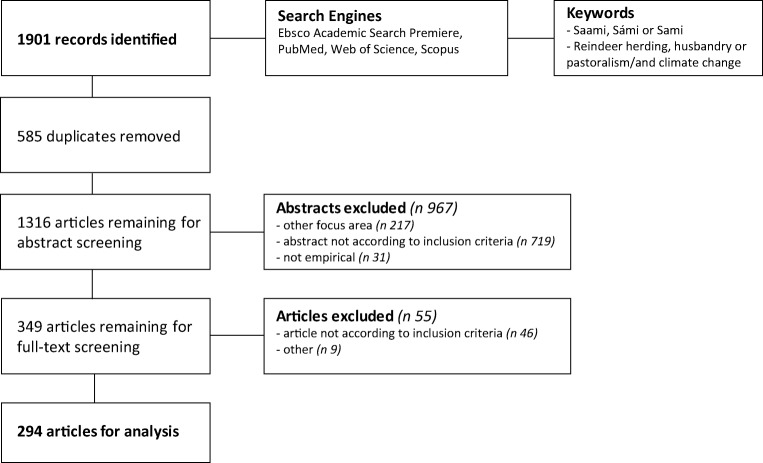
Flowchart of the systematic literature search

**Fig. 2:**
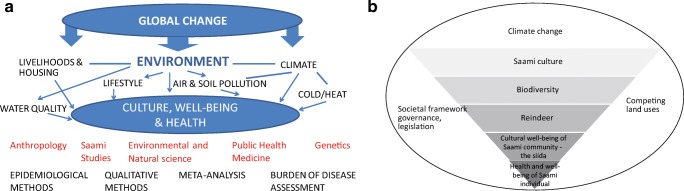
**a** Conceptual framework of the study. **b** Main factors influencing the health and well-being of Saami individual in reindeer-herding community

**Fig. 3:**
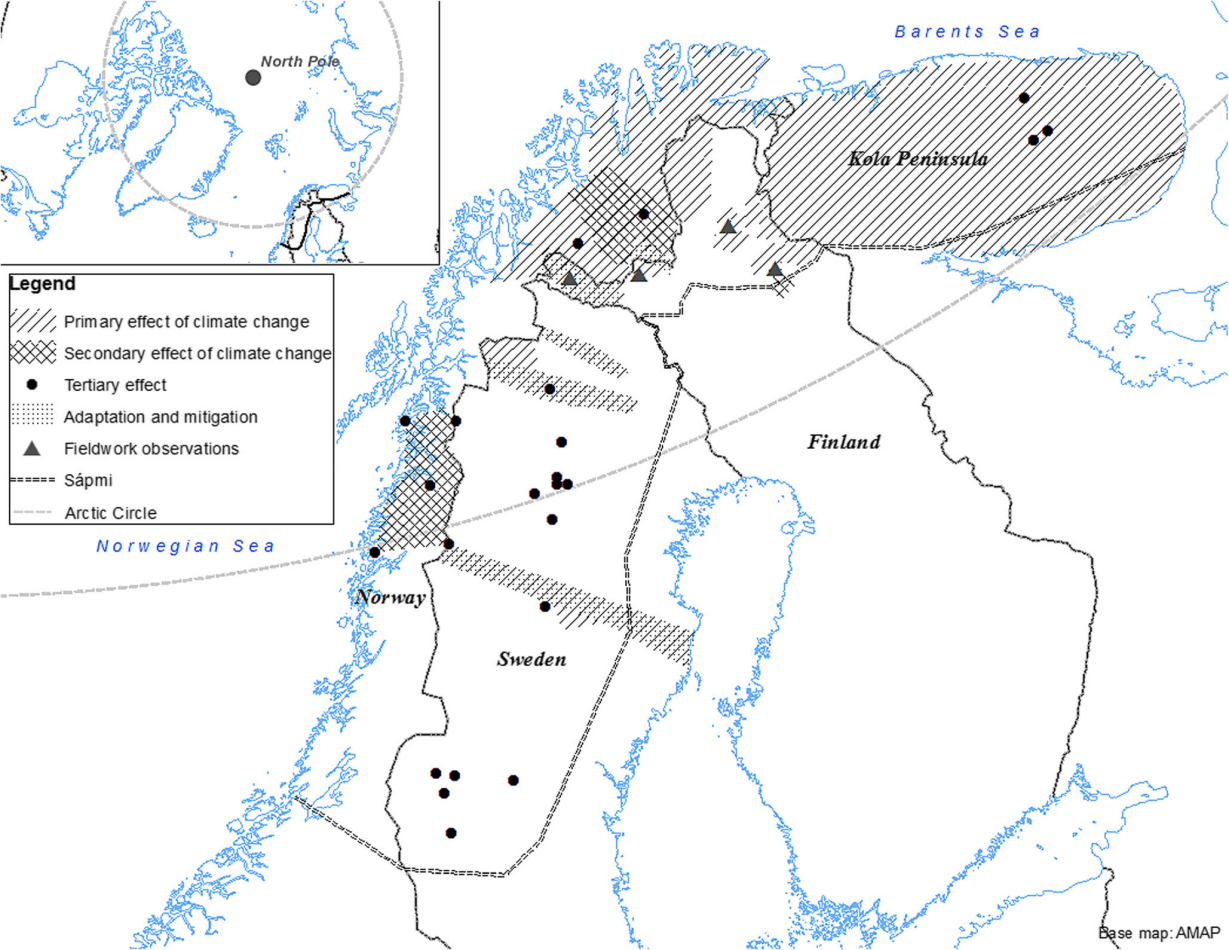
Local observations on the effects of climate change in Sápmi region based on the present systematic literature research and complementary fieldwork by Dr. Näkkäläjärvi

The main proposition is that the global change driven by the anthropogenic climate change is influencing entire cultures of the indigenous populations in the Artic (Fig. 1). We conceptualized the complex causal web schematically in Figs. 2 and 3. Figure 2a is an extension of the framework of the Center for Environmental and Respiratory Health Research multidisciplinary research program established in 2009 [11]. In the present form, “public health” appearing in the center was replaced with “culture, well-being, and health”. The arrows from Global change illustrate how the effects of global change are hypothesized to be mediated through climate, air, water, and soil pollution, housing, and life style. Figure 2a explains the multi-dimensional, direct and indirect effects of climate change to culture, well-being, and health.

We apply the proposition of Butler and Harley to divide the health effects of climate change into the primary, secondary, and tertiary effects. The primary effects arise from the direct changes and impacts of the physical system. The secondary effects arise due to alternations in the ecology of vectors, parasites, and host animals, and effects mediated by the allergens and air pollutants are included in this category. The tertiary effects refer to wider scale of effects caused by intersection of climate, politics, and ecology, both human and non-human [12••]. Our framework in the Saami context is expanded to cover cultural and social effects of climate change. Cultural and social effects are likely to influence health and well-being.

Figure 2a, b describes the different methodological approaches to understand the factors effecting Saami health and cultural well-being in a changing climate and the main forces driving the global change.

Figure 2a describes the central research disciplines and research methods that applied to study the holistic effects of climate change on Saami culture. Figure 2b describes the ethno-ecological niche [13, 14] of Saami people and the changing framework of their culture. The niche includes the individual and the *siida* that is kinship-based Saami communal structure that manages reindeer work in its own area. Each siida has its own pasture areas, but the siida system is very flexible [15]. The cultural ecological framework of the niche consists of the *eallu* (the reindeer) and biodiversity. Saami people and culture are not isolated but part of larger Saami community and national society with its legislation, practices, education system, and jurisprudence. Climate change has been identified as a new framework for Saami culture and livelihoods due to its far-reaching and holistic influences [16••, 17•].

### Literature Search and Complementary Evidence

We conducted a systematic literature review on peer-reviewed publications covering any articles on (1) Saami people; (2) Saami reindeer herding; and (3) climate change and its effects on the Saami and the Sápmi region. We focused only on studies providing empirical research data, either qualitative or quantitative. The inclusion criteria were that the article provided information on (1) Saami culture, health, well-being and/or livelihoods; and (2) the effects of climate change on the Sápmi and Saami livelihoods. Keywords were selected based on tests of different keywords in academic search engines. Articles published in 1990–2017 were included.

In addition to the systematic literature search, we included evidence from an ethnographic fieldwork conducted by Dr. Näkkäläjärvi, which included interviews of 30 reindeer herders from four Saami communities in Finland 2015–2017, during the conduct of this systematic review. These interviews provided important insight into the holistic effects of climate change perceived by reindeer herders and it influenced the development of the theoretical framework.

## Results

### Literature Search and Characteristics of Studies

Figure 1 present a flowchart of the search strategy. The primary search identified 294 studies, 126 conducted in Norway (43%), 83 in Sweden (28%), 51 in Finland (17%), only 12 covered Russia (4%), and 22 were multinational (8%). Table 1 provides an overview on main study topics. Most of the studies characterize the environment, culture, livelihoods, and health without any consideration of the role of climate change.

**Table 1:** Published articles on Saami health and well-being by the main topic and country, 1990–2017

Topic	Norway		Sweden		Finland		Russia		Total
	*n*	Publication year	*n*	Publication year	*n*	Publication year	*n*	Publication year	
General health	20	1999, 2002–2016	3	2010–2013	2	1997, 2002	1	1999	26
Cause-specific mortality	3	2006–2009	5	2004–2012	5	1995–1999, 2008	0		13
Occupational health	0	–	4	2004–2008, 2017	5	1991–1994, 2006	0		9
Asthma and allergies	2	1999 and 2002			1	1991	0		3
Cardiovascular diseases	4	1998, 2013–2014	2	2004–2008	3	1995–1997, 2001	0		9
Diabetes	3	1998, 2016–2017			0		0		3
Cancer	1	2005	3	2002, 2008–2014	3	2002, 2010–2012	0		7
Genetic diseases and genetics	9	1992–1994, 2002–2008	2	2004, 2008	3	1998–2001	4	1997–1999, 2008–2017	18
Mental health	27	1998, 2000–2017	7	2010–2017	1	1994	0		35
Dental health	0		4	2006–2014	0		0		4
Environmental exposures	2	1999, 2014			3	1999, 2003–2009	1	1999	6
Nuclear fallout and radiation	4	1996, 2000–2015	4	1990–1999, 2014	2	2005–2010	0		10
Diet and lifestyle	9	1999–03, 2007–2017	7	1999, 2004–2013	2	1995	1	2008	18
Substance use	7	1990, 2002–2011	2	2011, 2015	0				9
Cultural well-being	16	1996–1998, 2003–2017	6	2006–2015	3	1995, 2010–2016	0		25
Discrimination	7	2008–2015	2	2011–2012	0		0		7
Violence	3	2015–2017			0		0		3
Health services	15	2005–2013	1	2013	1	2012	0		17
TOTAL	135		52		34		7		

### Environment, Livelihoods, and Culture

**Fig. 4:**
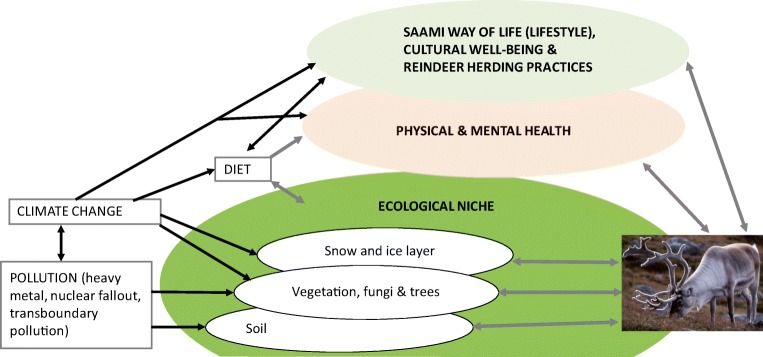
Simplified presentation on causal effects of climate change to the health and cultural well-being of Saami in Reindeer livelihood context

Traditional Saami settlement area includes both sub-arctic and arctic areas with boreal forest, coastal areas, and large mountain regions with alpine vegetation. Snow covers the Sápmi area 8 months a year, and plays a central role in the climatic, ecological, and hydrological processes, and in the way of life and reindeer herding [18]. Saami language has a precise classification system for snow and knowledge of snow is an important part of reindeer herder’s knowledge [19]. Reindeer is important for Saami culturally and reindeer herding is means to maintain traditions, language, and cultural identity in changing world. Reindeer herding, as other traditional Saami livelihoods constitute important part of Saami well-being (see Fig. 4). Currently, reindeer herding is one of the driving forces together with other herbivore on shaping the vegetation in Sápmi region [20]; however, in boreal area forestry and competing land uses, such as mining, infrastructure development, and tourism affect both vegetation and reindeer herding [21, 22]. Although reindeer forage over 300 different plants in addition to fungi, ground lichen is the main forage for reindeer in winter. Lichen heaths are in heavily grazed areas and in areas where forestry and reindeer grazing coexist degraded, but there are regional differences in grazing pressure [23–25]. Human activity influences the range selection of reindeer, and increase grazing pressure inside the reindeer-herding area [26, 27], but there is also evidence that reindeers can locally habituate towards human activities [28]. Predation influences significantly the mortality among reindeers, but the effects on the total reindeer demography and livelihood are debated. Losses are compensated for the reindeer herders [29, 30, 31•].

Saami reindeer herding is distinctively inherited profession and kin-ties are important for reindeer management. [32]. Reindeer herding is connected to Saami well-being so that the quality of life decrease if the livelihood is lost [33]. Women constitute a minority of full-time reindeer herders, being actively involved in reindeer-herding culture [34••]. Evidence from Sweden suggests that there are gender differences in the status of reindeer herder, and different expectations for women [35].

Saami reindeer herding has adopted new technology and methods such as supplementary feeding. It is argued, that these changes have increased the adaptive capacity of reindeer herding, but increased the dependence on subsidies [26, 36]. However, there are regional variations in different reindeer-herding models and in the use of new methods. Modernization has created social and economic pressures for the reindeer-herding communities. Small proportion of Saami reindeer herders is involved in tourism services, which provide better income and concurrently offers means to maintain traditional livelihood [36–38]. Governance and market economy have forced Saami to change their reindeer-herding model in Kola Peninsula. Industrialization has influenced Saami to abandon their culture [39, 40].

Assimilation measures have caused significant loss of Saami language; but language revitalization measures have improved the situation [8, 41]. The smallest Saami languages alive have only few speakers and it is likely that language loss will continue in the near future. Traditional Saami home regions are located in rural areas with sparse population. Outmigration to urban areas is an increasing trend, but the potential effects of outmigration to demography, Saami society social, and culture have not yet been explored. Migrants are characterized as young and mostly female [42]. Outmigration influences the communal viability, coherence, and vitality of the traditional Saami regions, and poses challenges to the cultural well-being of the migrants.

Saami and their livelihoods are vulnerable and exposed to socioeconomic changes and top-down governance. Adaptive capacity of Saami reindeer herding is limited geographically and in terms of governance [31•, 43]. Small population size and social and assimilative legacy pose serious challenges for the possibilities of Saami to maintain their culture and way of life in changing climate.

### Saami Lifestyle and Well-Being

It has been suggested that genetics together with Saami traditional lifestyle that includes significant physical activity and a healthy diet including reindeer meat, fish, and berries have positive effects on the Saami health reducing the risk of chronic diseases typically linked to western lifestyle [44–47]. Unfortunately, there is evidence that the lifestyle is changing: Saami experience less physical activity and traditional diet is, especially outside Saami core areas, being partly or completely replaced with western diet [48–51]. Saami women are in general more obese than women population in the same area. Saami men are less obese than the other population in the same area [52]. Obesity is a risk factor for pre-diabetes and diabetes mellitus. The findings indicate that if the cultural and socioeconomic changes continue among Saami population effecting the traditional lifestyle and diet, it is likely that diabetes mellitus will become a public health concern among Saami population. Change or loss in traditional diet also indicates change in cultural values, livelihoods, and lifestyle.

Recent studies found no differences in the substance use between the Saami and the majority population in Norway [53, 54] and Sweden [55, 56]. However, 50% of non-natural deaths among Saami reindeer herders in Sweden occurred under the influence of alcohol [57•].

In Northern Norway, the Saami were subjected to violence more than the general population; 22% Saami women experienced sexual violence [58]. Several findings from Sweden and Norway indicate that a major part of Saami population has experienced ethnic discrimination and it has negative implications to mental well-being and quality of life [59, 60]. Several important cultural and social factors, such as living in Saami core areas, involvement in reindeer herding, Saami as a native language, strong family ties, and communality, seem to protect Saami from mental health problems and provide resilience towards ethnic discrimination [60–62].

### Health and Well-Being

Several population-based epidemiologic studies have estimated cause-specific mortality and the occurrence of chronic diseases between the Saami and the majority population. Main results are summarized in Table 2. The Saami health is on average similar, or slightly better compared to the health of other populations in the same area in Finland, Norway, and Sweden [7••].

**Table 2:** Overview on the main results on Saami health and well-being

References	Study topic	Results	Discussion and trend
[44, 46, 47, 57]	Mortality	Specific cause mortality among Saami and non-Saami population in Sweden, Norway and Finland rather similar	Recent findings are missing, however there seems to be a trend that cause-specific mortality is starting to resemble general population
[44], Sjölander P, Hassler S and Janlert U. Stroke and acute myocardial infarction in the Swedish Sami population: Incidence and mortality in relation to income and level of education. Scand J Public Health 2008: 36(1): 84–91.Doi:10.1177/1403494807085305.		Reindeer-herding Saami men had a lower risk of death from cardiovascular and gastrointestinal diseases and cancer in Sweden	
[44] Norum J and Nieder C. Socioeconomic characteristics and health outcomes in Sami speaking municipalities and a control group in northern Norway. Int J Circumpolar Health 2012: 71: 19127. Doi:10.3402/ijch.v71i0.19127.	Life expectancy	Life expectancy greater among people living in Saami core areas in Norway compared with general population (females 79.5 and men 72.0 years). In Sweden, the life-expectance was similar among the Saami and non-Saami populations	
[34, 57], Hassler S, Sjolander P, Johansson R, Gronberg H and Damber L. Fatal accidents and suicide among reindeer-herding Sami in Sweden. Int J Circumpolar Health 2002: 63 Suppl 2: 384–388; Pekkarinen A. Changes in reindeer-herding work and their effect on occupational accidents. Int J Circumpolar Health 2006: 65(4): 357–364.Doi:10.3402/ijch.v65i4.18125. Sjolander P, Daerga L, Edin-Liljegren A and Jacobsson L. Musculoskeletal symptoms and perceived work strain among reindeer herders in Sweden. Occupational Medicine-Oxford 2008: 58(8): 572–579. Doi:10.1093/occmed/kqn153.	Occupational accidents and health in reindeer herding	Off-road traffic-related deaths and accidents are more common among Saami compared with general population	Off-road traffic is essential for reindeer-herding work. Although recent findings are missing, it seems that off-road related deaths are not increasing among Saami population.
		Other risks are musculoskeletal pain, mental stress and accidents in slaughtering.	Findings indicate that mental stress may become a serious concern for reindeer-herding livelihood.
Selnes A, Bolle R, Holt J and Lund E. Atopic diseases in Sami and Norse schoolchildren living in northern Norway. Pediatric Allergy and Immunology 1999: 10(3): 216–220. Doi:10.1034/j.1399-3038.1999.00032.x. Selnes A, Bolle R, Holt J and Lund E. Cumulative incidence of asthma and allergy in north Norwegian schoolchildren in 1985 and 1995. Pediatr Allergy Immunol 2002: 13(1): 58–63. Doi: 10.1034/j.1399-3038.2002.01009.x	Asthma and allergies	Prevalence of asthma and allergies is increased among the Saami children in Norway	Changes in traditional lifestyle and diet are likely to explain the trend. Trend is alarming, since atopic diseases and asthma are major causes of morbidity.
[7]	Cardiovascular disease	No significant differences in occurrence of cardiovascular disease among Saami in Norway. Reindeer herding in Sweden Saami show lower incidences of cardiovascular diseases than other Saami.	The prevalence of cardiovascular diseases is effected by way of life and diet and Saami with western lifestyle have higher risk for cardiovascular diseases.
Broderstad AR and Melhus M. Prevalence of metabolic syndrome and diabetes mellitus in Sami and Norwegian populations. The SAMINOR - A cross-sectional study. BMJ Open 2016: 6(4). Doi::10.1136/bmjopen-2015-009474. Naseribafrouei A, Eliassen B, Melhus M and Broderstad AR. Ethnic difference in the prevalence of pre-diabetes and diabetes mellitus in regions with Sami and non-Sami populations in Norway - the SAMINOR1 study. Int J Circumpolar Health 2016: 75(1): 31697. Doi:10.3402/ijch.v75.31697. Edin-Liljegren A, Hassler S, Sjölander P and Daerga L. Risk factors for cardiovascular diseases among Swedish Sami--a controlled cohort study. Int J Circumpolar Health 2004: 63 Suppl 2: 292–297.	Diabetes mellitus and pre diabetes	No ethic differences in the prevalence of diabetes mellitus among Saami compared with other populations in the same area in Norway.	There are regional differences and livelihood-related risks on the relative risk and prevalence of diabetes mellitus among Saami population.
Hassler S, Soininen L, Sjolander P and Pukkala, P. Cancer among the Sami--a review on the Norwegian, Swedish and Finnish Sami populations. Int J Circumpolar Health 2008: 67(5): 421–432. Doi:10.3402/ijch.v67i5.18351.	Cancer	Overall risk of cancer does not differ significantly from the general population. Saami on Sweden have higher risk for ovarian cancer, Skolt Saami in Finland and Swedish Saami have higher risk for stomach cancer	
Harbo, H. F., Utsi, E., Lorentzen, Å. R., Kampman, M. T., Celius, E. G., Myhr, K. et al., Low frequency of the disease-associated DRB1*15-DQB1*06 haplotype may contribute to the low prevalence of multiple sclerosis in Sami. Tissue Antigens, 2007: 69: 299–304. doi:10.1111/j.1399-0039.2007.00803.x	Neurological diseases	Prevalence of MS lower among Saami than in Norwegian population	
[46, 57, 63] Silviken A, Haldorsen T and Kvernmo S. Suicide among Indigenous Sami in Arctic Norway, 1970–1998. Eur J Epidemiol 2006: 21(9): 707–713. Doi:10.1007/s10654-006-9052-7	Mental health	Increased risk of suicide among the Saami men in Sweden, Norway, and Finland. Mental health of Saami adolescents and Saami in reindeer herding is a concern. In general, risk for suicide among Saami adolescents is slightly elevated compared with other population. However, there are no ethnic differences on actual suicide attempts.	Findings indicate that loss of traditional livelihoods, Saami language, living outside Saami core areas and socioeconomic pressure from surrounding society seem to expose Saami to mental health problems. Findings indicate that the trend is increasing.
Alinaghizadeh H, Tondel M and Walinder *R.* Cancer incidence in northern Sweden before and after the Chernobyl nuclear power plant accident. Radiat Environ Biophys 2014: 53(3): 495–504. Doi:10.1007/s00411-014-0545-6.; Mehli H, Skuterud L, Mosdøl A and Tønnessen A. The impact of Chernobyl fallout on the Southern Saami reindeer herders of Norway in 1996. Health Phys 2000: 79(6): 682–690.	Nuclear fallout	Main source of 137Cesium exposure for Saami is reindeer meat and parts. The epidemiologic studies rule out any major effect of the nuclear fallout on cancer incidence among the exposed population. However, the prevention measures have effected the diet, economy, social life and livelihoods of Saami	Levels are significantly reduced also in most contaminated areas in Sápmi.
[48–50]	Diet and lifestyle	Saami traditional diet and lifestyle seem to protect Saami from lifestyle diseases	Findings indicate that Saami lifestyle and diet is changing to resemble the majority population. Change or loss in traditional diet also indicates change in cultural values, livelihoods and lifestyle
[55, 56]	Substance use	There are no significant ethnic differences on the use of substances	
[52, 61, 64].	Cultural well-being	Strong Saami ethnic identity seems to be important for physical and mental health of Saami.	Cultural well- being of Saami is associated with environmental relationship, traditions, livelihoods, Saami language, living in Saami core areas, social network and kin.
[58, 60–62]	Discrimination and violence	Saami have been subjected to discrimination and violence more than general population	However, Saami with strong ethnic identity have high resilience towards discrimination.
Daerga L, Sjölander P, Jacobsson L and Edin-Liljegren A. The confidence in health care and social services in northern Sweden – a comparison between reindeer-herding Sami and the non-Sami majority population. Scandinavian Journal of Public Health 2012: 40(6): 516–522. Doi:10.1177/1403494812453971; Norum J and Nieder C.Socioeconomic characteristics and health outcomes in Sami speaking municipalities and a control group in northern Norway. Int J Circumpolar Health 2012: 71: 19127. Doi:10.3402/ijch.v71i0.19127; Nystad T, Melhus M and Lund E. Sami speakers are less satisfied with general practitioners’ services. Int J Circumpolar Health 2008: 67(1): 114–121.Doi: 10.3402/ijch.v67i1.18246	Access to social and health services and implications to health outcome	The findings indicate that the Saami have equal access to social and health services and similar health outcomes compared with reference population	However, Saami speakers have reported lower satisfaction and communication difficulties in health services

Unfortunately, the findings also indicate that cultural and language loss among Saami population due to assimilation policies produces mental and social problems among Saami and the burden is passed on to future generations [65]. The studies have also indicated that living outside Saami core area, loose connection to Saami livelihoods and culture and loss of Saami language can increase mental health problems and decrease resilience towards ethnic discrimination [61, 62]. Saami adolescents living outside Saami core areas use more practitioner’s services than Saami in the core area or peer living in the same area [66]. This indicates that Saami are willing to seek help for their problems and living outside Saami core areas brings health challenges for Saami. A new emerging threat for Saami mental health is hopelessness and fear for the future identified in a qualitative study among Saami youth. Loss of Saami livelihood, weakened possibilities to maintain Saami culture and poor legal status are estimated to pose a serious mental health risk and impact on suicidal behavior [63••].

Living in Saami core areas, involvement in reindeer herding, Saami as a native language, strong family ties and communality, and possibility to maintain Saami culture and livelihoods increase Saami well-being [60–62, 64, 66].

### Primary Effects of Climate Change

Studies on the primary effects of climate change were focused on ongoing and past changes in the vegetation and climate and the climate scenarios (see Fig. 3) in Sápmi. Local observations by reindeer herders and studies confirm that climate change has already altered conditions in Sápmi region. The most prominent are changes in snow cover and vegetation. The ethnographic interviews by Dr. Näkkäläjärvi provided evidence that observations on these changes are common and consistent among reindeer herders. Shrub expansion is occurring rapidly in tundra and mountain range, and the tree line is moving north- and upwards [67, 68].

It is estimated that the days with snow cover are significantly shortened, but regional differences can be important. Winter thaws, rain-on-snow and refreezing events that are projected to increase creating ice crust [18, 69], and have a negative effect on reindeer pasture conditions, since reindeer cannot dig nutrition underneath ice cover. Climate change increases the risk of wildfires that have negative health effects and result loss of pasture land. Climate change is projected to threaten the lichen ecosystems in high latitudes and increases competition with other vegetation, especially vascular plants [67], but on the other hand, the predicted warming will decrease the cold period and days with snow cover increasing utilization of other vegetation for forage [69]. Predicted warming will increase heat stress among humans and reindeer. Changes in precipitation, increased heat stress, insect harassment, and changes in snow conditions in winter have an effect on predation and reindeer population dynamics [69, 70]. It is predicted that productivity of reindeer forage will probably increase, but the nutritive value and quality could decrease [67].

Weather-related accidents were predicted to either decrease [18] or increase [71]. Based on the interviews, the risk for accidents has increased among reindeer herders. Changes in the carrying capacity of ice, snow quality, and formation are likely to increase the risk for transport-related accidents and in the mountain region risk for avalanches can increase. Based on available knowledge, the primary health effects of climate change are likely to increase among Saami reindeer herders. Saami reindeer-herding models differ regionally and vary from semi-nomadic to local, and effects of climate change and adaptation possibilities thus vary significantly. Based on available evidence, it is not possible to estimate how the climate change influences different reindeer-herding models. Climate change is estimated to have both negative and positive effects for reindeer herding. However, the net balance is likely to vary significantly regionally depending on geography, local climate, environment, adaptation and mitigation measures, competing land uses, social system, and Saami population, reindeer-herding models, governance, and socioeconomic factors.

### Secondary Effects of Climate Change

Our search identified only a few studies on the secondary effects of climate change. Northern-Atlantic Oscillation (NAO) produces mild winter weather that creates difficult pasture conditions for reindeer. The NAO incidents are expected to increase in future and climate is likely to become more variable in Sápmi region [69]. The NAO incidents have a negative effect to the winter survival of reindeer. Based on evidence from fieldwork, the increased NAO incidents have contributed to the changes in reindeer-herding practices. Warm winters will promote the development of animal parasite outbreaks among reindeers and parasite species are moving northward [72]. Based on available evidence, the most prominent secondary effects are increased outbreaks of animal-borne diseases, indications are that milder weather and less snow cover promote the spread of new diseases northwards [73–75]. Warm winters also promote the survival of geometrid moths that is one of the main drivers for vegetational change in Sápmi. Increase in precipitation increases erosion and risks for accidents.

Arctic snow cover is a storage for contaminants and heavy metal pollution and reduced snow day cover and increased precipitation can expose humans to these pollutants [18].

### Tertiary Effects

The effects of climate change on the environment and livelihood are likely to influence the future expectations, which may induce mental health problems. Climate change had created fear for future and together with socioeconomic and governance pressure these can be overwhelming. The fear is focused on the future of Saami culture and way of life and disappearance of cultural knowledge and traditions. Herders have reported on increased stress, anxiety, worrying, and depression [16••, 31•, 35, 76, 77].

Climate change adaptation and opening new resources can limit the adaptation possibilities in Sami reindeer herding by diminishing pastures. Hydropower development has influenced significantly Saami reindeer herding and culture [78] and climate change is likely to change conditions more favorable for hydropower development [18]. Wind power development is already affecting the range selection of reindeer and to the rights of Saami [27, 79] and increased temperatures will increase the productivity of boreal forest and expansion of boreal forest to new areas creating land-use competition and opening new possibilities for forestry.

### Adaptation and Mitigation

Climate adaptation and mitigation were approached in the identified studies from two perspectives: (1) adaptation in reindeer herding to the adverse effects of climate change and (2) reindeer as a tool for climate adaptation. We could not identify any studies that discussed how the whole Saami culture and other livelihoods could adapt to the climate change. Findings indicate that adaptation possibilities are limited and available measures are flexible use of pasture land, use of vast environmental knowledge, herding, provide nutrition with supplementary feeding, or fell trees to gain reindeer access to arboreal lichen. Human-animal relationship and reindeer-herding traditions form a basis for adaptation measures [31•, 43, 80]. There is evidence that adaptation measures have cultural effects. Reindeer herders fear that the use of supplementary nutrition changes profoundly their cultural livelihood [81], and similar concerns have been raised on the use of GPS-technology on reindeer [82]. Based on fieldwork data, supplementary feeding and GPS use are means for adaptation in difficult conditions, but they have also important cultural effects and may erode the cultural knowledge of the reindeer herder, which can reduce the adaptive capacity of reindeer-herding culture in the future. Reindeer become tamer due to the use of supplementary nutrition and it affects the range selection of the reindeers.

Reindeer grazing can mitigate the effects of climate change, especially in tundra. Reindeer grazing can protect the tundra biome from shrubification and tree line encroachment [68, 83] . Incentive grazing in summer increases surface albedo, delays snowmelt and decreases the ground from heating in snowmelt season [84••]. Reindeer in fact is a key species for the environment and Saami inhabiting the area.

## Discussion

**Fig. 5:**
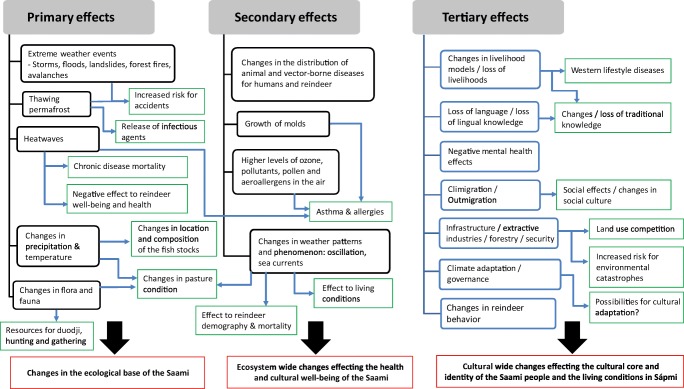
Main factors affecting health and well-being of Saami and Sápmi in the changing climate based on available data and application of theoretical framework

Figure 5 summarizes our analysis of the main factors affecting health and well-being of Saami and Sápmi in the changing climate based on available data and application of theoretical framework (Fig. 2a).

### Environmental and Cultural Change

A recently published meta-analysis suggests that climate change is having profound disruptive effects at local levels and that local observations can make an important contribution to understanding the pervasiveness of climate change on ecosystems and societies [17•]. Indigenous communities from the Arctic have reported that weather conditions have been less predictable in the past decades [85, 86]. Our findings and fieldwork support these observations. The environmental changes affecting flora and fauna are projected to continue in the Arctic (Table 3) [87].

**Table 3:** Key literature and results on primary, secondary and tertiary effects of climate change to Sápmi region and Saami people

Article	Primary effects	Secondary effects	Tertiary effects	Mitigation	Adaptation
Dobler et al. Regional climate change projections for the Barents region. Earth System Dynamics Discussions: 2016: 1–22. DOI:10.5194/esd-2016-27.	Projections on temperature, cloudiness and precipitation in the Barents region				
Furberg et al. Facing the limit of resilience: perceptions of climate change among reindeer herding Sami in Sweden. Global Health Action 2011: 4: 1–11. DOI:10.3402/gha.v4i0.8417.	Perception of primary effects of climate change to winter conditions		Adverse mental health and cultural effects of climate change		Flexibility and technology means for adaptation
Lof A. Examining limits and barriers to climate change adaptation in an Indigenous reindeer herding community. Clim Dev 2013: 5(4): 328–339. DOI:10.1080/17565529.2013.831338.	Perception of primary effects of climate change to winter conditions				Governance limits possibilities for adaptation
Pape R. and Loffler J. Climate change, land use conflicts, predation and ecological degradation as challenges for reindeer husbandry in northern Europe: what do we really know after half a century of research? Ambio 2012: 41(5): 421-434DOI:10.1007/s13280-012-0257-6	Projected effects of climate change to conditions relevant for reindeer herding			Adaptation possibilities	
Parkinson A.J. and Evengård B. Climate change, its impact on human health in the Arctic and the public health response to threats of emerging infectious diseases. Global Health Action 2009: 2: 88–90.	Risk for accidents and health threats from extreme weather events	Potential changes in vector-borne and infectious diseases	Adverse mental health effects and diet and lifestyle changes increasing risk for lifestyle diseases		
Tryland M. Are we facing new health challenges and diseases in reindeer in Fennoscandia? Rangifer 2012: 32(1): 35–47.	Effects of changing climate to reindeer demography, pastures and condition	Reindeer parasite and diseases outbreaks		Adaptation in reindeer herding with supplementary feeding and technology	
Vowles et al. Expansion of deciduous tall shrubs but not evergreen dwarf shrubs inhibited by reindeer in Scandes mountain range. Journal of Ecology 2017: 105(6): 1547–1561. Doi:10.1111/1365-2745.12753.	Effects of climate change to vegetation (shrubification)			Herbivore is potentially of great importance for shaping arctic shrub expansion and its associated ecosystem effects	
Eliasson et al. Identification of development areas in a warming Arctic with respect to natural resources, transportation, protected areas, and geography. Futures 2017: 85: 14–29. Doi:10.1016/j.futures.2016.11.005.			Opening of new development areas in warming climate		
Willox et al. Examining relationships between climate change and mental health in the Circumpolar North. Reg Environ Change 2015: 15(1): 169–182. DOI:10.1007/s10113-014-0630-z.			Climate change is likely an emerging mental health challenge for Circumpolar Indigenous populations		
Cohen et al. Effect of reindeer grazing on snowmelt, albedo and energy balance based on satellite data analyses. Remote Sens Environ 2013: 135: 107–117 Doi: /10.1016/j.rse.2013.03.029.				Summer reindeer herding can delay snowmelt, increase surface albedo and to decrease the ground heating in the snowmelt season.	

The melting sea ice and opening of new resources for extraction [88] increase the risk for environmental accidents and are likely to increase land-use competition limiting the possibilities for Saami to adapt to the climate change and possibilities to maintain their traditional livelihoods.

The most important traditional game for the Saami is willow ptarmigan (*Lagopus lagopus*). A recent study suggests that predation-ptarmigan interactions have changed, most likely due to climate change. Ptarmigans are vulnerable to the temperature fluctuations, increased precipitation, and population declines are likely to occur in changing climate [89]. Fishing is important to both inland and coastal Saami, and especially in coastal area fishing is a viable livelihood [90]. It is projected that changes in temperature effect the movement, location, and demography of fish stock [91]. However, how these changes are likely to influence Saami fishing culture, is not known.

### Health Perspectives

Saami are considered to be highly adapted to Arctic conditions [92] but it is possible that socioeconomic and lifestyle changes have negative effects on adaptive capacity. Our analysis suggests that the lifestyle, diet, and morbidity of the Saami is changing to resemble the majority populations posing threats for both the physical and mental health of the Saami, increasing general morbidity, and making them more vulnerable to the adverse health effects of climate change. Changes in lifestyle and diet expose Saami to cardiovascular disease. Increased climate change-related heat exposure is associated with an increase in cardiac events and especially elderly are vulnerable [93]. We have identified that the livelihood changes may have direct effects on the physical health and cultural well-being of the Saami, which need to studied further and taken into consideration in governance.

Projected changes in temperature are likely to increase the risk of adverse secondary health risks both to Saami and reindeer. Disease outbreaks of pathogens transmitted by ticks and voles could emerge in previous non-endemic geographic areas [73, 94]. Infectious diseases are likely to have more outbreaks in changing climate and expand to new territories into the north [74, 95]. Increase in precipitation and flooding events could create conditions favorable for mold growth in homes and this may result in an increase in mold-related disorders and allergies. A new emerging health threat especially in Russian Arctic is the melt of permafrost and release of pathogens [96].

According to AMAP human health report, human exposure to most POPs and metals is declining across many parts of the Arctic but interactions between climate change and contaminant transport have the potential to change human exposure in the Arctic significantly [97].

A major contributor to mental health among Arctic indigenous populations is chronic psychosocial stress linked to rapid socioeconomic development and this development is expected to accelerate due to climate change [98, 99]. Our findings support these observations.

Climate adaptation and mitigation actions such as wind power or hydropower development may increase the stress and mental pressure among the Saami and increase pasture competition. Therefor, climate adaptation and mitigation measures should take into account possible effects to the health and well-being of Saami.

Saami are indigenous people and international covenants obligate the states to protect Saami culture and way of life for future. Health and well-being are basic human rights but in indigenous context culturally meaningful life is a human rights question. Currently there is not sufficient information to appropriately assess health and cultural well-being among the Saami, and therefor, it is challenging to evaluate whether international human rights obligations are met and to what extent adaptation to climate change will be culturally sustainable. Thus, evaluation and long-term monitoring of Saami health and cultural well-being is crucial. Development of a monitoring system is urgent. This study has elaborated the existing knowledge on the relations between environment, culture and health and on the influences of climate change to culture and health of Saami, and shown that the evidence base is limited.

Saami communities around Sápmi face many socioeconomic challenges that threaten their possibilities to maintain their culture and identity. These multi-stress factors weaken the possibilities of Saami to adapt to climate change and maintain their culture. According to the fieldwork data, climate change poses risk for reindeer health and health of reindeer herders in terms of increased risks for accidents and future health prospects. Most prominent health effects are the mental health effects: increased stress, concern over the future of Saami way of live and pressures to abandon traditional Saami lifestyle. Also literature search support these findings.

Our analysis suggests that climate change brings both environmental, economic, cultural and social effects on reindeer herding as economic livelihood and as cultural way of life.

Comparison of the health and well-being of Saami to the health of other indigenous people is possible only at a general level. Comprehensive population-based studies and registry-based data are missing—in Nordic countries ethnic registries are forbidden—and hence, even the definition of a Saami is being disputed. The most comprehensive overview on the health of indigenous and tribal people was published in 2016. The study used general indicators—such as life expectancy and infant mortality—available from registries, excluding morbidity and mental health. The analysis shows that direct comparison of health of indigenous peoples is not possible, because of different status and legal interpretation of indigenousness, differences in data collection methods and indicators and lack of standardization of different methods [100]. However, it can be argued that based on available evidence, the health of Saami is relatively good or better when compared on a general level to other indigenous peoples globally, but overview on cultural well-being and metal health of Saami is missing. More research and development of a monitoring system are needed to understand the health and cultural well-being of Saami in changing climate taking into consideration Saami values and traditions and major drivers for change.

## Conclusion

The multidisciplinary methodology presented here to theorize and understand how climate change effects all aspect of Saami life is central for monitoring the effects and finding ways for culturally sustainable adaptation. Cultural well-being in the Saami context depends on social community and kinship structure, environmental relationship, and traditional livelihoods, and ultimately, possibility to maintain Saami ethnicity and language. Loss of language, culture, and living in urban areas expose Saami to lifestyle changes that can have negative implications for mental and physical health.

Saami have been a study object for centuries in different disciplines, nonetheless holistic analysis is missing on the status and capacities of Saami people to survive and adapt in changing climate. The warming climate and new insight call for a new, comprehensive assessment of the effects of climate change to Sápmi and Saami culture and projected outlook for the future. However, climate change has already influenced Saami culture substantially. Outmigration can in future change to climigration and have a profound effect on the Saami culture and viability of traditional Saami home region. Saami population is small and number of Saami that speak Saami as native language is even smaller. Societal changes and assimilation policies have contributed the loss of language and cultural knowledge and made Saami people even more vulnerable to the negative and cumulative effects of climate change.

A crucial challenge for the future of Saami people and vulnerability among the Saami is the small population size, dispersed settlement, and urbanization that limits the possibilities for cultural adaptation in the changing climate.

## Footnotes

1 In this study we use the term lifestyle according to Saami understanding. It refers to, inter alia, Saami way of life, values, social structure, traditions, and customs. It includes the tradition of Saami livelihoods, identity, and lingual heritage and habitation pattern.
